# Expression of the aryl hydrocarbon receptor (AhR) in facial melasma skin compared to healthy perilesional skin^☆^

**DOI:** 10.1016/j.abd.2025.501181

**Published:** 2025-08-13

**Authors:** Carolina Nunhez da Silva, Tony Fernando Grassi, Hélio Amante Miot, Ana Cláudia Cavalcante Espósito

**Affiliations:** a Department of Infectious Diseases, Dermatology, Imaging Diagnosis and Radiotherapy, Faculty of Medicine, Universidade Estadual Paulista, Botucatu, SP, Brazil; b Laboratório GENOTOX, Faculty of Medicine, Universidade Estadual Paulista, Botucatu, SP, Brazil; c Faculty of Medicine, Universidade do Oeste Paulista, Presidente Prudente, SP, Brazil

Dear Editor,

Melasma is a chronic, acquired hypermelanosis, most prevalent in adult women with intermediate skin types (III to V), especially in intertropical countries such as the Middle East, India, and Latin America.^1^, ^2^

In the last decade, its prevalence has increased in Brazil.^3^ A study that evaluated the volume of searches on the Google platform demonstrated a 54.4% global increase in interest in the topic since 2000; there is an association with the country's sunlight exposure, oral contraceptive use, and carbon dioxide emissions.^4^

The aryl hydrocarbon receptor (AhR) is a cytoplasmic protein that, when activated, undergoes nuclear translocation and acts as a regulatory transcription factor for the response to environmental stimuli and maintenance of cell homeostasis. It is expressed in various skin cell types, including keratinocytes, melanocytes, fibroblasts, sweat ducts, and hair follicles, participating in processes such as cell proliferation, differentiation, apoptosis, inflammation, and pigmentation.^5^, ^6^

AhR can be activated by polychlorinated biphenyls, dioxins, or other polycyclic aromatic hydrocarbons (PAHs) present in environmental pollution, as well as endogenous metabolites induced by ultraviolet radiation. Activated AhR upregulates enzymes involved in the clearance of xenobiotics (such as CYP1A1, CYP1A2, CYP1B1, and UGT1A6). The activity of these enzymes results in the production of reactive oxygen species (ROS), inducing p38, which increases the activity of MITF (Microphthalmia-Associated Transcription Factor), regulating tyrosinase expression in melanocytes.^5^, ^6^, ^7^

Air pollution plays an important role in extrinsic aging. Synergistically with sun exposure and genetic factors, it may be a risk factor for the onset and maintenance of melasma pigmentation, due to the cutaneous penetration of PAHs and activation of AhR.^8^ Since, to date, no study has evaluated the role of AhR in melasma, an investigation was conducted to compare its differential activation in melasma and adjacent healthy skin.

After approval by the ethics committee of the Botucatu School of Medicine - Unesp (n. 6,590,628), 20 adult women with facial melasma who had not been treated for more than 30 days underwent two skin biopsies (3 mm): one from the skin with facial melasma and the other from clinically healthy, adjacent skin, up to 2 cm from the lesion border.

After being paraffin embedded, the 40 specimens were stained with hematoxylin & eosin and submitted to immunohistochemistry (DAB) for Ki67 (clone MIB1, Dako, M7240, Glostrup, Denmark – 1/50) and for AhR (clone EPR7119(N)(2), ab190797, Abcam, Cambridge, MA – 1/100). All immunohistochemical reactions were performed manually, validated in the tissues indicated by the antibody package inserts, and titrated to the lowest concentration to avoid background staining (Supplementary Material).

The main outcome was the number of stained epidermal nuclei per x400 field, analyzed blindly regarding the topography (melasma or healthy skin). The entire epidermal sample was evaluated and counted for all participants. The staining of pilosebaceous units was also evaluated. The thickness of viable epidermis was assessed using three measurements in interfollicular areas. The epidermal proliferation rate was calculated using the HSCORE score of Ki67 nuclear staining. The HSCORE is a semiquantitative method for evaluating the immunohistochemical expression of proteins in the cell nucleus. It weights the percentage of stained cells and the intensity of staining (0 = absent, 1 = weak, 2 = moderate, 3 = strong).^9^

The variables were compared between topographies using a linear mixed-effects model, and significance was defined as p < 0.05. The sample size was based on the expectation of a difference between topographies of more than 10% of expression among the sampled skins (alpha 0.05 and beta 0.2).

The clinical and demographic data of the sample are shown in Table 1, and none of the participants were smokers. Immunohistochemical staining (Figure 1, Figure 2) revealed higher nuclear AhR expression in the melasma epidermis compared with the adjacent healthy skin (1.3 vs. 1.1 nuclei/field; p = 0.03). There was no cytoplasmic AhR staining in the epidermis. In the pilosebaceous units, both nuclear and cytoplasmic staining were observed (Fig. 3), being more frequent in melasma than in the adjacent healthy skin (70% vs. 30%; p = 0.03). There was staining in the eccrine sweat gland ducts, in both topographies (p > 0.9).

**Table 1:** Main clinical and demographic information of female participants (n = 20 women).

Variables	Values
Age (years), mean (SD)	44.9 (9.2)
Phototype, n (%)	
II	8 (40%)
III	5 (25%)
IV-V	7 (35%)
Pregnancy history, n (%)	
Never	3 (15%)
1-2	11 (55%)
3 or more	6 (30%)
Use of oral contraceptives, n (%)	1 (5%)
Daily sun exposure, n (%)	7 (35%)
Family history of melasma, n (%)	15 (75%)
Age of onset of melasma, mean (SD)	33.3 (10.8)
mMASI, mean (SD)	4.0 (2.4)

mMASI, Modified Melasma Area and Severity Index.

**Figure 1:**
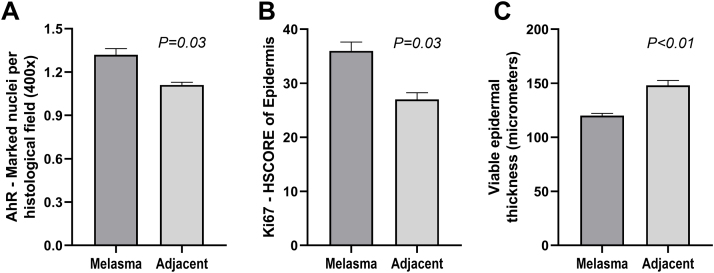
Comparative graphs of AhR nuclear expression in the epidermis (A), Ki67 score (B) and epithelial thickness (C) with melasma and adjacent healthy skin (n = 20).

**Figure 2:**
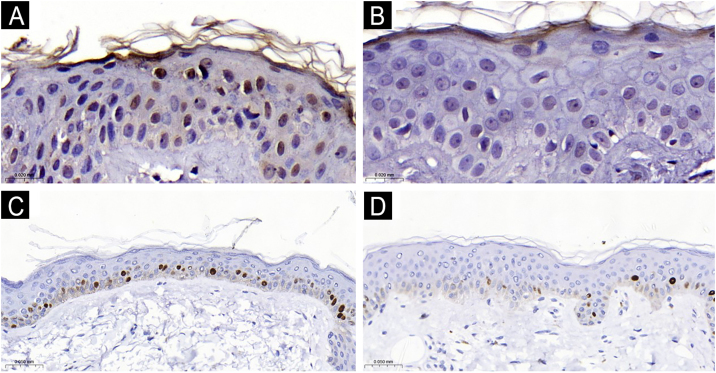
Epidermal nuclear expression of AhR (×1000): A. Melasma. B. Adjacent skin. Epidermal nuclear expression of Ki67 (×400): C. Melasma. D. Adjacent skin.

**Figure 3:**
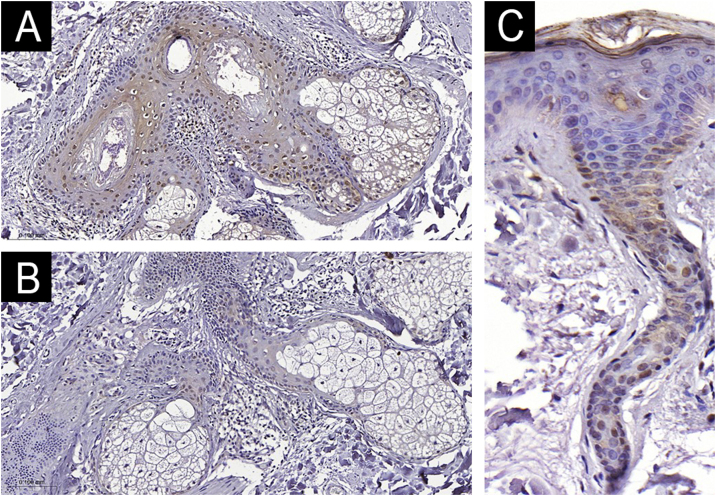
AhR expression in the pilosebaceous follicle (×200). A. Melasma. B. Adjacent skin. C. AhR expression in the eccrine sweat gland ducts (×400).

The epidermis with melasma (Figure 1, Figure 2) showed thinner epithelial thickness (187 vs. 207 μm; p < 0.01), but a higher proliferation rate (HSCORE 36 vs. 27; p = 0.02). There was no correlation between HSCORE, mMASI, epidermal thickness, and epithelial AhR expression (p > 0.3).

The higher expression of AhR in the epidermis and pilosebaceous units in melasma may indicate the involvement of environmental stimuli (especially pollution) in its pathogenesis and contribute to the higher prevalence of melasma in urban centers. Furthermore, ultraviolet light, particularly UVB, and exposure to visible light generate formylindole-(3,2-*b*)-carbazole, a high-affinity ligand and endogenous transcriptional activator of AhR, reinforcing the synergistic action of solar radiation on atmospheric pollutants.^10^

Microparticulate pollutants penetrate the skin through intracellular, intercellular pathways, and through hair follicles and sweat glands. In melasma, there is impairment of the skin barrier and a reduction in epithelial thickness compared to the adjacent skin, which may favor the permeation of pollutants.^2^ ROS generated by AhR activation contributes to oxidative damage to the epidermis and upper dermis. This inflammatory microenvironment accelerates collagen degradation and the secretion of inflammatory mediators that can induce persistent melanogenesis.^2^, ^5^, ^7^

AhR activation can mediate melanogenesis via the canonical or non-canonical pathway. In the canonical pathway, upon activation, AhR translocates to the nucleus, where it interacts with its heterodimerization partner ARNT (Aryl Hydrocarbon Receptor Nuclear Translocator), forming a complex that binds to specific DNA response elements, such as the xenobiotic response element. This results in the transcription of genes such as *CYP1A1* and *B1*, which influence melanin synthesis. The non-canonical pathway involves direct or indirect interactions of AhR with signaling proteins, independently of ARNT, including activation of intracellular kinases and pathways such as MAPK (Mitogen-Activated Protein Kinase). These pathways can promote the expression of pro-melanogenic factors, such as MITF, which induce melanin synthesis.^5^, ^6^, ^7^, ^8^, ^11^ However, such melanogenic pathways have been demonstrated in cell cultures and murine models, and the present experiment was based on immunohistochemistry of paraffin-embedded tissue from dermatosis pigmentosa.

In addition to melanocytic hypertrophy, melasma shows structural alterations of the entire epidermis, upper dermis, and barrier function. Increased cell replication rates with thinner epithelial thickness may reflect the intense cell transit induced by growth and inflammatory factors in the upper dermis, which is structurally compromised in the disease. A similar phenomenon occurs in the atrophic epithelium of photoaging.^2^, ^12^

The main limitations of this study are the modest sample size, slight differential staining, the inclusion of women only, and the lack of a functional study of the pathways activated by AhR. Furthermore, the use of manual immunohistochemistry with an experimental antibody can be considered a limitation. Further studies should explore the effect of AhR blockade on the suppression of melanogenesis and skin aging, as well as investigate possible melanogenic stimuli originating from the sebaceous glands in melasma.^13^

In conclusion, greater expression of AhR was observed in the epidermis and pilosebaceous units of melasma skin compared to adjacent skin, suggesting a role in its pathogenesis. The melasma epidermis also showed smaller thickness and a higher replication rate.

## Financial support

Research Award - Exposome Grant 2022 Vichy.

## Authors’ contributions

Carolina Nunhez da Silva: Design and planning of the study; analysis and interpretation of data; drafting and editing of the manuscript; critical review of the literature; critical review of the manuscript; approval of the final version of the manuscript.

Tony Fernando Grassi: Analysis and interpretation of data; drafting and editing of the manuscript; collection of data; approval of the final version of the manuscript.

Hélio Amante Miot: Design and planning of the study; analysis and interpretation of data; statistical analysis; drafting and editing of the manuscript; critical review of the literature; critical review of the manuscript; approval of the final version of the manuscript.

Ana Cláudia Cavalcante Espósito: Design and planning of the study; analysis and interpretation of data; drafting and editing of the manuscript; critical review of the literature; critical review of the manuscript; approval of the final version of the manuscript.

## Conflicts of interest

None declared.

## Research data availability

The entire dataset supporting the results of this study was published in this article.

## Scientific Associate Editor

Luciana P. Fernandes Abbade.
