# Supplementary material for: Expression of the aryl hydrocarbon receptor (AhR) in facial melasma skin compared to healthy perilesional skin
Source: An Bras Dermatol. 2025 Aug 13;100(5):501181. doi: 10.1016/j.abd.2025.501181 (PMC12361975; doi:10.1016/j.abd.2025.501181)
Supplement: Supplementary file 1 [file mmc1.docx]

**MATERIAL SUPLEMENTAR.**


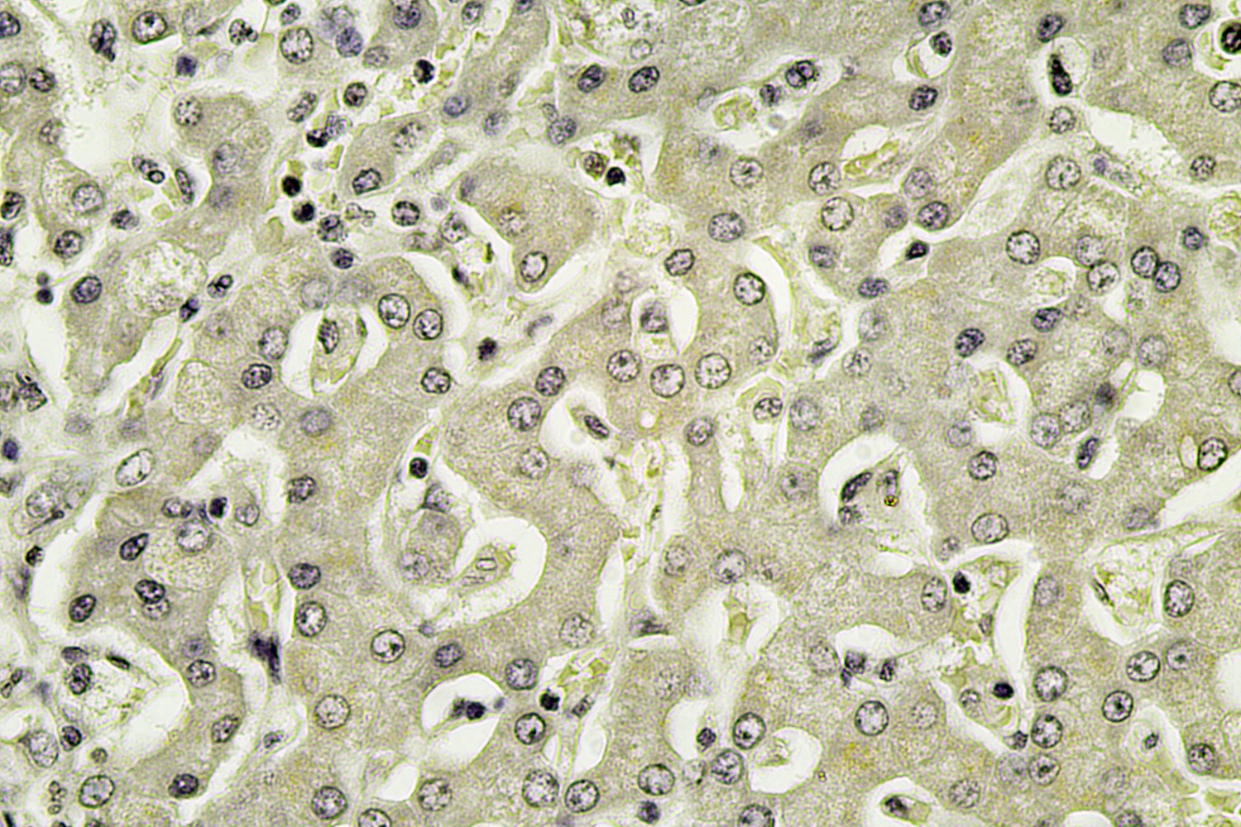


**Figura S1.** Controle positivo da marcação citoplasmática do AhR em fígado humano (400x).


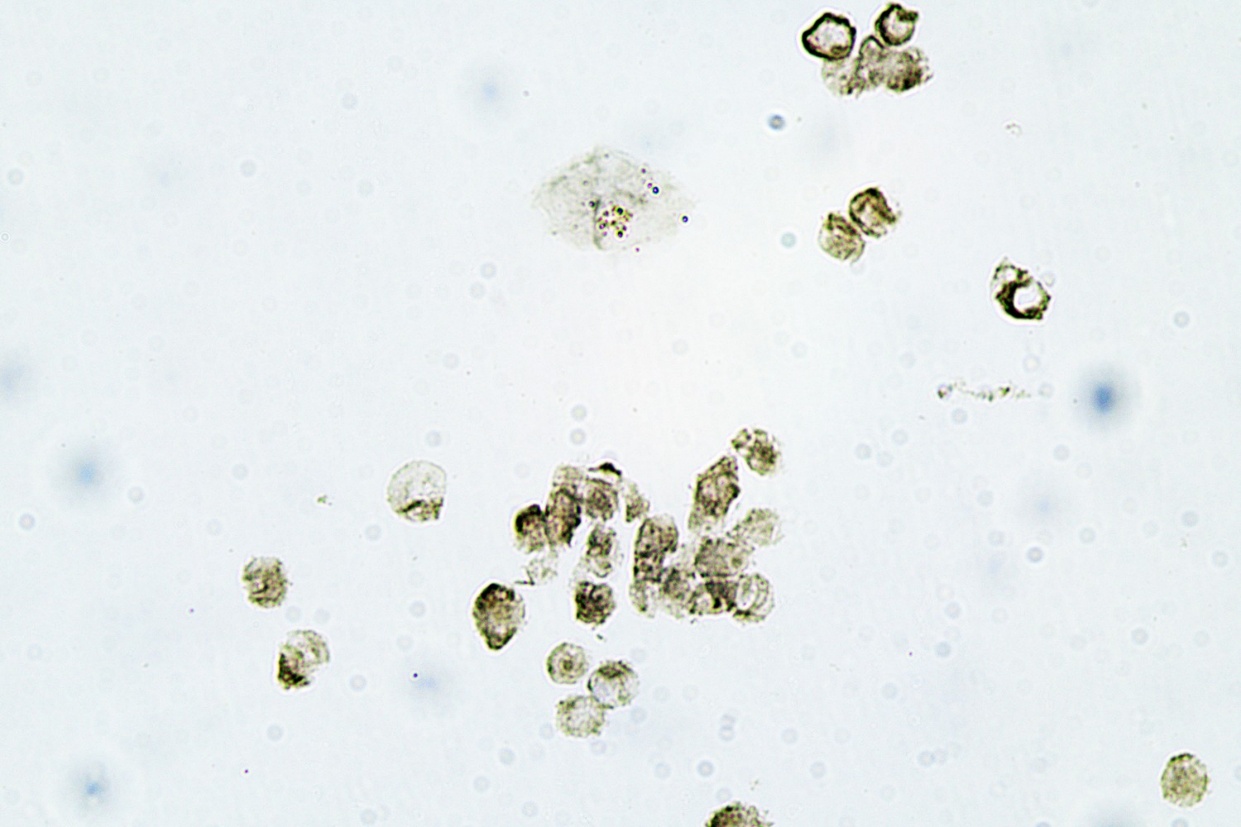


**Figura S2.** Controle positivo da marcação nuclear do AhR (1/100) em células HeLa (400x).


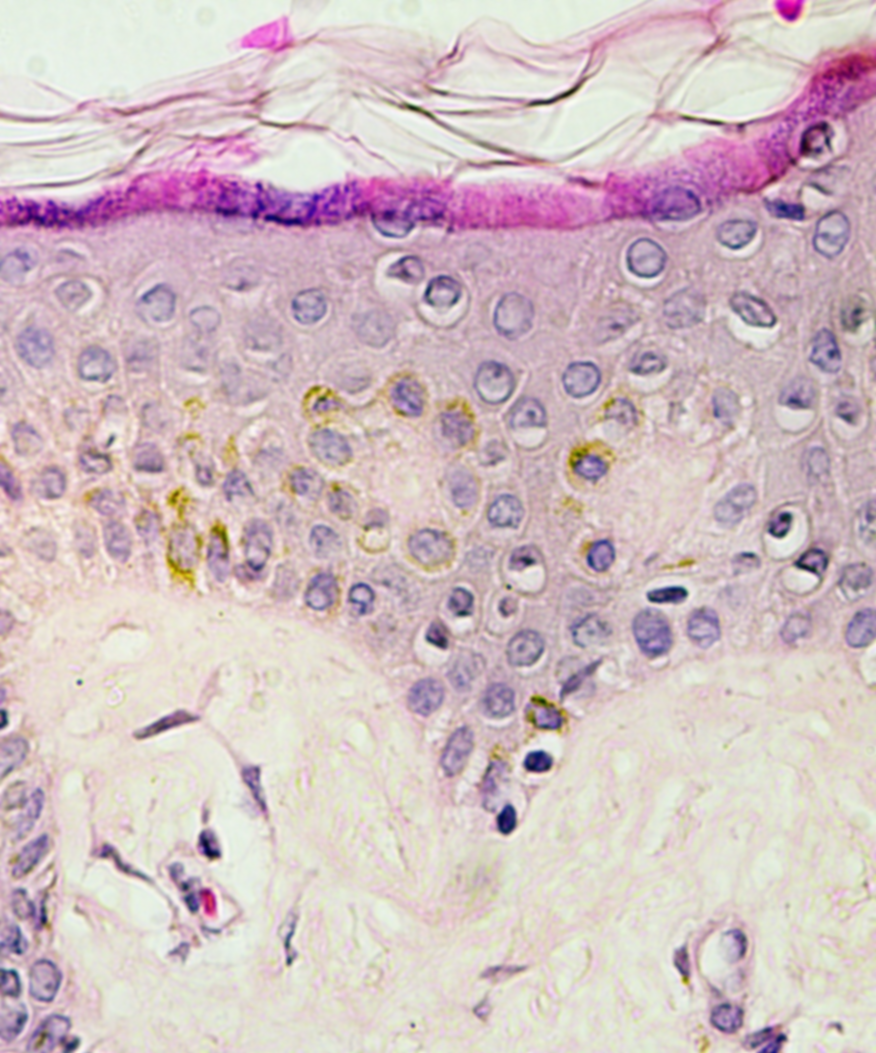

**Figura S3.** Corte de pele de melasma, referente ao caso apresentado na figura 2A (H&E; 1000x).


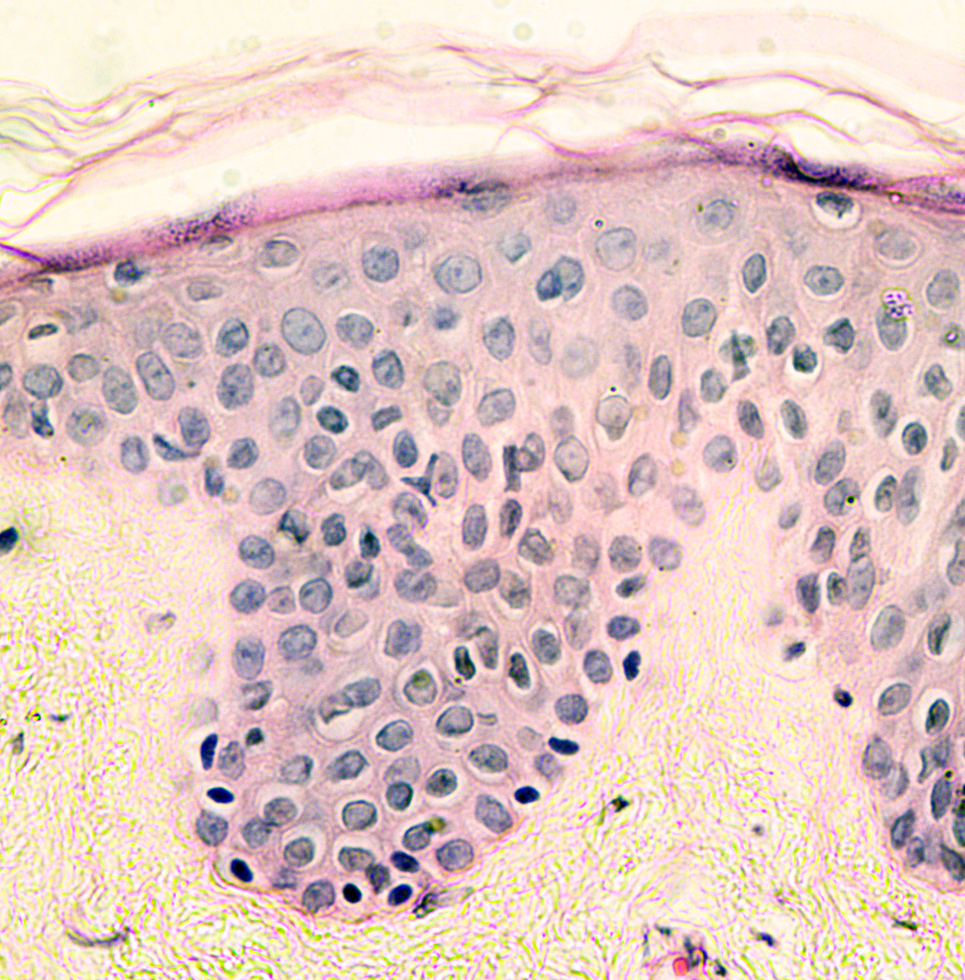


**Figura S4.** Corte de pele sã adjacente, referente ao caso apresentado na figura 2B (H&E; 1000x).
